# Electrospun Active Biopapers of Food Waste Derived Poly(3-hydroxybutyrate-*co*-3-hydroxyvalerate) with Short-Term and Long-Term Antimicrobial Performance

**DOI:** 10.3390/nano10030506

**Published:** 2020-03-11

**Authors:** Kelly J. Figueroa-Lopez, Sergio Torres-Giner, Daniela Enescu, Luis Cabedo, Miguel A. Cerqueira, Lorenzo M. Pastrana, Jose M. Lagaron

**Affiliations:** 1 Novel Materials and Nanotechnology Group, Institute of Agrochemistry and Food Technology (IATA), Spanish National Research Council (CSIC), Calle Catedrático Agustín Escardino Benlloch 7, 46980 Paterna, Spain; kjfigueroal@iata.csic.es; 2 International Iberian Nanotechnology Laboratory (INL), Avenida Mestre José Veiga, 4715-330 Braga, Portugal; danielamenescu@gmail.com (D.E.); miguel.cerqueira@inl.int (M.A.C.); lorenzo.pastrana@inl.int (L.M.P.); 3 Polymers and Advanced Materials Group (PIMA), Universitat Jaume I (UJI), Avenida de Vicent Sos Baynat s/n, 12071 Castellón, Spain; lcabedo@uji.es

**Keywords:** PHBV, essential oils, inorganic nanoparticles, biopapers, electrospinning, migration, active packaging

## Abstract

This research reports about the development by electrospinning of fiber-based films made of poly(3-hydroxybutyrate-*co*-3-hydroxyvalerate) (PHBV) derived from fermented fruit waste, so-called bio-papers, with enhanced antimicrobial performance. To this end, different combinations of oregano essential oil (OEO) and zinc oxide nanoparticles (ZnONPs) were added to PHBV solutions and electrospun into mats that were, thereafter, converted into homogeneous and continuous films of ~130 μm. The morphology, optical, thermal, mechanical properties, crystallinity, and migration into food simulants of the resultant PHBV-based bio-papers were evaluated and their antimicrobial properties were assessed against *Staphylococcus aureus* (*S. aureus*) and *Escherichia coli* (*E. coli*) in both open and closed systems. It was observed that the antimicrobial activity decreased after 15 days due to the release of the volatile compounds, whereas the bio-papers filled with ZnONPs showed high antimicrobial activity for up to 48 days. The electrospun PHBV biopapers containing 2.5 wt% OEO + 2.25 wt% ZnONPs successfully provided the most optimal activity for short and long periods against both bacteria.

## 1. Introduction

The packaging industry has increased the demand of active materials obtained from renewable resources due to the environmental concerns related to the extensive use of conventional plastics and also to the consumer requests for safer, more nutritious, and high-quality food [1]. Polyhydroxyalkanoates (PHAs) are bio-based and biodegradable aliphatic polyesters of high potential to replace polyolefins in packaging applications [2]. PHAs are synthesized in the mitochondria of a wide range of Gram-negative (G−) and Gram-positive (G+) microorganisms [3]. They can be divided into short chain length (scl-PHAs) with monomers with three to five carbon atoms and medium chain length (mcl-PHAs) with monomers with more than six carbons [4]. Among them, scl-PHAs are the most studied, including the poly(3-hydroxybutyrate) (PHB or P3HB) homopolyester and its copolymer poly(3-hydroxybutyrate-*co*-3-hydroxyvalerate) (PHBV). The latter shows lower crystallinity and melting point as well as greater flexibility, which broadens its industrial applicability [4,5]. In order to confer active properties to PHAs, several substances can be incorporated into the biopolymers such as essential oils (EOs) and inorganic or metal nanoparticles (MNPs) [6,7].

EOs are the product of the secondary metabolism of plants, separated from the aqueous phase, which is formed by various volatile components such as terpenes, alcohols, acids, esters, epoxies, aldehydes, ketones, amines, and sulphides, among others [8]. This wide range of compounds are responsible for the strong biological activity and also the antioxidant, antimicrobial, antifungal, and antiviral properties of plants [9]. In addition, EOs are biologically safe and have a low risk of causing resistance in pathogens [10]. In addition, they are classified as Generally Recognized as Safe (GRAS) by the U.S. Food and Drug Administration (FDA) [11]. Among them, oregano essential oil (OEO) is one of the most important and it is widely used in the pharmaceutical, food, and cosmetics industries [12]. It comes from the genus *Origanum* that belongs to the *Lamiaceae* family [13], which is constituted by more than 38 monoterpenoids [14]. Their main active compounds are carvacrol, thymol, p-cymene, and γ-terpinene, which are responsible for its high antimicrobial and antioxidant activities [15,16]. These active compounds have been particularly related to the inhibition of G− bacteria such as *Escherichia coli* (*E. coli*), *Pseudomonas aeruginosa* (*P. aeruginosa*), and *Salmonella typhimurium* (*S. typhimurium*) and G+ bacteria including *Staphylococcus aureus* (*S. aureus*), *Listeria monocytogenes* (*L. monocytogenes*), *Bacillus subtilis* (*B. subtilis*), *Streptococcus pyogenes* (*S. pyogenes*), and *Alicyclobacillus acidoterrestris* (*A. acidoterrestris*) [12,15,17,18,19]. Some other studies have also demonstrated the high antioxidant activity of OEO, which ranges from 80% to 90% inhibition of free radicals of 2,2-diphenyl-1-picrylhydrazyl (DPPH) and 2,2′-azino-bis-(-3-ethylbenzothiazoline-6-sulfonic acid) (ABTS) [12,20,21] due to the presence of hydroxyl groups (–OH) in its chemical structure [22].

Zinc oxide (ZnO) is an inorganic material, which can have up to three crystal structures including hexagonal wurtzite, cubic zinc-blende structure, and a rarely-observed cubic rock-salt (NaCl-type) [23]. The wurtzite form is the most thermodynamically stable structure in environmental conditions. The zinc-blende structure is metastable while the cubic rock-salt structure is stable under extreme pressure [24]. Particles of ZnO are the fourth most used metal particles in the world after those of iron, aluminum, and copper. It is commonly used in sunscreens and also in the food industry, which presents some advantages over other metallic substances due to its high mechanical stability, thermal stability at an ambient temperature, biocompatibility, and low cost and toxicity [25,26]. ZnO is also among the five zinc compounds categorized as GRAS by the U.S. FDA (21CFR182.8991) [27,28,29]. Zinc oxide nanoparticles (ZnONPs) can be synthesized by mechanochemical processing, sol-gel methods, and spray pyrolysis [30,31]. The antimicrobial effect of ZnONPs is exerted by different mechanisms of action such as reactive oxygen species (ROS), ion release (Zn^2+^), membrane dysfunction, nanoparticle penetration, interruption, and blockage of transmembrane electron transport. ZnONPs cause irreversible damage as they disintegrate the membrane and increase its permeability [32,33,34]. Numerous investigations have reported a broad spectrum of ZnONPs bactericide in most G− and G+ bacteria such as *E. coli*, *Salmonella enteritidis* (*S. enteritidis*), *S. typhimurium*, *Proteus vulgaris* (*P. vulgaris*), *Klebsiella pneumonia* (*K. pneumoniae*)*, S. pyogenes, Aeromonas hydrophila* (*A. hydrophila*), *B. subtilis, S. aureus, L. monocytogenes, P. aeruginosa, Enterococcus faecalis* (*E. faecalis*), *Sarcina lutea* (*S. lutea*), and more [35,36,37,38,39,40].

The combined use of such organic-inorganic substances can offer several advantages in the development of novel active materials. For instance, a good dispersion of ZnONPs in an EO-containing PHA matrix can be effectively achieved and the properties of the resultant composite are improved [41]. From a chemical point of view, the integration of MNPs in polymers can be attained by a stable incrustation of the nanoparticles in the polymer matrix without having any chemical interaction or an anchorage of the nanoparticles on the surface by means of covalent, ionic, or hydrogen bonds [42]. Additionally, this combination can improve the mechanical properties, thermal stability, conductivity, and chemical resistance of polymers. Their composites can also show antimicrobial and antioxidant properties for different periods of time [43,44,45]. Therefore, they can contribute to the design of active packaging that extends the shelf life of the products by a mechanism that delays or inhibits the microbial, enzymatic, and oxidative reactions, which are the main causes of food deterioration [46,47]. Then, the resultant packaging can serve to improve the distribution logistics and food preservation [48]. For instance, Al-Jumaili et al. [49] developed nanocomposite films combining renewable geranium essential oil (GEO) with ZnO. These sustainable nanocomposite films were proposed as active coatings of medical devices. In another study, Sani et al. [50] studied the effect of the combination of Melissa essential oil (MEO) and ZnO in chitosan composite films. The resultant composite showed a great deal of potential as antimicrobial and biodegradable films in food packaging applications. Ahmed et al. [51] also developed antimicrobial films incorporating ZnONPs in combination with clove essential oil (CEO) into polylactide/polyethylene glycol/poly(ε-caprolactone) (PLA/PEG/PCL) blends by solution casting. The films showed antibacterial efficacy for 21 days of storage at 4 °C against *S. aureus* and *E. coli* inoculated in scrambled egg. Heydari-Majd et al. [45] also developed active packaging films based on polylactide (PLA) containing ZnONPs and the *Zataria multiflora* Boiss essential oil aimed to extend the shelf life of meat products.

In this regard, the electrospinning technology offers the option to form high-performance active and bioactive systems based on polymer yarns of nanofibers with high surface-to-volume ratios by the application of high electrical fields to polymer solutions [52]. The resultant electrospun materials containing the active-releasing agents can then be incorporated into multilayer structures as coatings [53] or interlayers, [54] having a potential application in antimicrobial systems [55]. Furthermore, the application of annealing a thermal post-treatment below the biopolymer’s melting temperature (*T_m_*) allows us to obtain continuous and highly transparent films of more interest in the field of packaging [56]. Our previous research works have previously demonstrated that electrospun PHA films, that is, biopapers, containing either neat OEO [57,58] or ZnONPs [59], can exert a strong antimicrobial activity against different bacterial strains. The term “biopapers” refers to fiber-based materials made of natural polymers that are non-fiber based in origin such as PHAs. This is similar to conventional papers that refer to fiber-based materials made of cellulose or nanopapers to those made of nanocellulose. Moreover, the active packaging toward traditional packaging (total “inert” packaging) is designed to add valuable properties to foodstuff (e.g., extending the shelf life of foodstuff). Nevertheless, there are serious challenges faced by its commercialization due to the possible migration of the active additive from food packaging into foodstuff, and whose effects on consumer health are unknown [60].

In this context, the objective of this research was to assess the potential of the electrospinning technology to develop active biopapers of PHBV containing mixtures of OEO and ZnONPS to achieve both short-term and long-term strong antimicrobial performance. Initially, both antimicrobial agents were characterized and the content of ZnONPs in PHBV was optimized. Then, the electrospun hybrid PHBV biopapers were characterized in terms of their morphology, optical, thermal, and mechanical properties, and crystallinity. Thereafter, the antimicrobial activity of the electrospun biopapers was evaluated against strains of *E. coli* and *S. aureus* in an open and closed system for 48 days and, compared to equivalent biopapers, based on neat OEO or ZnONPs. Lastly, the amount of ZnONPs that might migrate from the electrospun PHBV biopapers into different food simulants was analyzed.

## 2. Materials and Methods

### 2.1. Materials

PHBV with a molar fraction of 3-hydroxyvalerate (HV) of ~20% was produced at the Universidade NOVA (UNOVA) de Lisboa (Lisbon, Portugal) at a pilot-plant scale. The copolyester was produced by means of mixed microbial cultures fed with fermented fruit that was supplied by SumolCompal S.A. (Lisbon, Portugal) as a waste from manufacturing fruit juice. Further details about the preparation of this PHA can be found elsewhere [61].

OEO, purity >99% and a relative density of 0.925–0.955 g/mL, was provided by Gran Velada S.L. (Zaragoza, Spain). ZnONPs (CR-4FCC1), 99% purity, specific surface of 4.5 m^2^/g, bulk density of 40 lb/ft^3^, and specific gravity of 5.6 were obtained from GH Chemicals LTD^®^ (Saint Hyacinthe, QC, Canada).

Methanol, HPLC grade with 99.9% purity and chloroform, reagent grade with 99.8% purity, were both obtained from Panreac S.A. (Barcelona, Spain). 1-butanol, reagent grade with 99.5% purity, and DPPH were both obtained from Sigma-Aldrich S.A. (Madrid, Spain). Ethanol absolute (≥ 99.9% vol.) was supplied by Honeywell^®^ (Frankfurt, Germany). Acetic acid glacial (99%) was supplied by Fisher Chemical^®^ (Loughborough, UK). Zinc standard for calibration of ICP (TraceCERT^®^, 1000 mg/L zinc metal of high-purity quality prepared with 2% nitric acid suitable for trace analysis and high-purity water, 18.2 MΩ.cm, 0.22 µm filtered) and nitric acid (70 vol%) were supplied by Sigma-Aldrich (Darmstadt, Germany).

### 2.2. Preparation of the Solutions for Electrospinning

The PHBV solution for electrospinning was prepared by dissolving 10% (wt/vol) of the biopolymer in a mixture of chloroform/1-butanol 75:25 (vol/vol) at room temperature. A solution containing OEO at 10 wt% in PHBV was also produced in the same conditions based on our previous research work [57]. To select the optimal concentration of ZnONPs in PHBV, different solutions with contents of 1 wt%, 3 wt%, 6 wt%, and 10 wt% of ZnONPs in PHBV were prepared. The formulations based on the combination of OEO and ZnONPs were tested by preparing PHBV solutions with 7.5 wt% OEO + 0.75 wt.% ZnONPs, 5 wt% OEO + 1.5 wt% ZnONPs, and 2.5 wt% OEO + 2.25 wt% ZnONPs.

### 2.3. Preparation of the Electrospun Biopapers

All the PHBV solutions containing OEO and ZnONPs were electrospun, in the same conditions, in a Fluidnatek^®^ LE500. A high-throughput electrospinning/electrospraying pilot line was manufactured and commercialized by Bioinicia S.L. (Valencia, Spain). Electrospining was performed by means of a 24 emitter multi-nozzle injector that was scanning vertically onto a metallic plate. The process conditions during electrospinning consisted of a flow-rate of 6 mL/h per single emitter, a voltage of 17 kV, and a tip-to-collector distance of 20 cm.

The resultant electrospun mats were subjected to annealing in a 4122-model press from Carver, Inc. (Wabash, IN, USA). The thermal post-treatment was performed at 125 °C, for 15 s, without pressure, based on previous conditions [57]. The resultant film samples, known as biopapers, had an average thickness of approximately 80–130 µm.

### 2.4. Characterization

#### 2.4.1. Thickness

The thickness of all the biopapers was measured, prior to testing, using a digital micrometer S00014 from Mitutoyo, Corp. (Kawasaki, Japan) with an accuracy of ±0.001 mm. Five different points of the samples were measured including one in the middle and two in each end.

#### 2.4.2. Morphology

Scanning electron microscopy (SEM), by means of a Hitachi S-4800 (Tokyo, Japan), and transmission electron microscopy (TEM), using a JEOL 1010 (Peabody, MA, USA), were used to determine the particle shape and size (diameter) distributions of ZnONPs and of the morphology of the electrospun PHBV fibers and their corresponding biopapers containing OEO and ZnONPs. For analyzing the dispersion of the ZnONPs in the electrospun PHBV fibers, the fibers were collected on a sandwich-type holder (Agar Scientific-G230, Agar Scientific Ltd., Essex, UK) with a mesh size of 3.05 mm. For the SEM cross-section observations, the biopapers were cryo-fractured by immersion in liquid nitrogen. The samples for SEM analysis were previously sputtered with a gold-palladium mixture for 3 min under vacuum. An accelerating voltage of 10 kV and a working distance of 8–10 mm was used during SEM analysis. For TEM, an acceleration voltage of 100 kV was employed. The size distribution of the particles and average fiber diameters was determined via ImageJ software using, at least, 20 microscopy images.

#### 2.4.3. Transparency

The light transmission of the biopapers was determined using an UV–Vis spectrophotometer VIS3000 from Dinko, Instruments (Barcelona, Spain). To this end, the absorption of light at wavelengths between 200 nm and 700 nm was measured in specimens sized 50 mm × 30 mm. Transparency (T) and opacity (O) were determined using Equations (1) and (2), respectively [62].

(1) $$T=\frac{A_{600}}{L}$$

(2) $$O=A_{500}L$$

where *L* is the biopaper thickness (mm), while $A_{500}$ and $A_{600}$ are the absorbances at 500 and 600 nm, respectively.

#### 2.4.4. Color

Color of the biopapers was determined with a Chroma Meter CR-400 from Konica Minolta (Tokyo, Japan). The color difference (ΔE*) was calculated using Equation (3) [62].

(3) $$\Delta E^{*}={\left[{\left(\Delta L^{*}\right)}^{2}+{\left(\Delta a^{*}\right)}^{2}+{\left(\Delta b^{*}\right)}^{2}\right]}^{0.5}$$

where ΔL*, Δa*, and Δb* corresponded to the differences between the brightness and color parameters of the PHBV biopapers containing the active substances and the values of the neat PHBV biopaper. The color change was evaluated according to the following criteria [63]: the difference was unnoticeable if ΔE* < 1. Only an experienced observer can notice the difference for ΔE* ≥ 1 and < 2. An unexperienced observer notices the difference when ΔE* ≥ 2 and < 3.5. A clear noticeable difference is noted if ΔE* ≥ 3.5 and < 5, and the observer notices different colors when ΔE* ≥ 5. Tests were performed in triplicate.

#### 2.4.5. X-Ray Diffraction Analysis

Wide angle X-ray diffraction (WAXD) was performed on the biopaper samples using a Bruker AXS D4 Endeavour diffractometer from Bruker Corporation (Billerica, MA, USA). The samples were scanned, at room temperature, in reflection mode using incident CuK_α_ radiation (*k* = 1.5406 Å), while the generator was set up at 40 kV and the filament current was set at 40 mA. The data were collected over the range of scattering angles (2*θ*) comprised in the 5–40° range. Peak fitting was carried out using Igor Pro software package and Gaussian function was used to fit the experimental diffraction profiles obtained.

#### 2.4.6. Thermal Analysis

The thermal properties were studied by thermogravimetric analysis (TGA) in a thermobalance TGA/STDA851e/LF/1600 from Mettler Toledo Inc (Schwarzenbach, Switzerland) under nitrogen atmosphere. After conditioning the samples in the sensor for 5 min at 30 °C, the samples were heated from 25 °C to 700 °C at a heating rate of 10 °C/min. The first derivative of thermogravimetry (DTG) curves, expressing the weight loss rate as the function of time, were also obtained using TA analysis software. All the tests were carried out in triplicate.

#### 2.4.7. Mechanical Tests

Tensile tests were performed on an Instron Testing Machine Model 4469 from Instron Corp (Canton, MA, USA) according to ASTM Standard D 638. The biopapers were dumbbell-shaped to 115 mm × 16 mm. The cross-head speed was fixed at 10 mm/min. Four samples were tested for each biopaper and the average values of the mechanical parameters and standard deviations were reported.

#### 2.4.8. Antimicrobial Activity

*S. aureus* CECT240 (ATCC 6538p) and *E. coli* CECT434 (ATCC 25922) strains were obtained from the Spanish Type Culture Collection (CECT, Valencia, Spain). The bacterial strains were stored in phosphate buffered saline (PBS) with 10 wt% tryptic soy broth (TSB) obtained from Conda Laboratories (Madrid, Spain) and 10 wt% glycerol at −80 °C. Previous to each study, a loopful of bacteria was transferred to 10 mL of TSB and incubated at 37 °C for 24 h. A 100-µL aliquot from the culture was again transferred to TSB and grown at 37 °C to the mid-exponential phase of growth. The optical density showing an absorbance value of 0.20 and measured at 600 nm in a UV–Vis spectrophotometer VIS3000 from Dinko, Instruments (Barcelona, Spain) determined that the initial bacterial concentration was approximately a 5 × 10^5^ colony-forming unit (CFU)/mL.

The values of minimum inhibitory concentration (MIC) and minimum bactericide concentration (MBC) of the OEO and ZnONPs were tested against the two selected food-borne bacteria following the plate micro-dilution protocol described in the “Methods for Dilution Antimicrobial. Susceptibility Tests for Bacteria That Grow Aerobically; Approved Standard Tenth. Edition (M07-A10)” by the Clinical and Laboratory Standards Institute (CLSI). During this test, a 96-well plate with an alpha numeric coordination system (columns 12 and rows A-H) were used, where 10 µL of the tested samples were introduced in the wells with 90 µL of the bacteria medium. In the wells corresponding to A, B, C, E, F, and G columns, different concentrations of ZnONPs and OEO (0.078, 0.156, 0.312, 0.625, 1.25, 2.5, 5, 10, 20, and 40 µg/mL) were tested, in triplicate, from rows 1 to 10. Columns D and H were used as a control of ZnONPs and OEO in TSB without bacteria. Rows 11 and 12 were taken as the positive control, that is, only TSB, and a negative control, that is, *S. aureus* and *E. coli* in TSB, respectively. The plates were incubated at 37 °C for 24 h. Thereafter, 10 µL of resazurin, a metabolic indicator, was added to each well and incubated again at 37 °C for 2 h. Upon obtaining the resazurin change, the wells were read through a color difference. The MIC value was determined as the lowest concentration of OEO and ZnONPs presenting growth inhibition.

The antimicrobial performance of the electrospun PHBV biopapers was determined based on the guidelines of the Japanese Industrial Standard JIS Z2801 (ISO 22196:2007) for film samples [64]. The dimension of the biopapers was 1.5 cm × 1.5 cm. Onto the PHBV biopapers containing OEO and ZnONPs (test films) and PHBV biopaper without OEO and ZnONPs (negative control film), a microorganism suspension of *S. aureus* and *E. coli* was applied. Thereafter, the inoculated samples were placed in open bottles and incubated for 24 h at 24 °C and at a relative humidity (RH) of at least 95%. Bacteria were recovered with PBS, 10-fold serially diluted, and incubated for 24 h at 37 °C to quantify the number of viable bacteria by a conventional plate count. The antimicrobial activity reduction (*R*) was evaluated from 1 (initial day), 8, 15, 22, 30, 40, and 48 days using Equation (4).

(4) $$R=\left[Log\left(\frac{B}{A}\right)-Log\left(\frac{C}{A}\right)\right]=Log\left(\frac{B}{C}\right)$$

where *A* is the average of the number of viable bacteria on the control sample immediately after inoculation, *B* is the average of the number of viable bacteria on the control sample after 24 h, and *C* is the average of the number of viable bacteria on the test sample after 24 h. The next assessment was followed to evaluate the antibacterial activity of the biopapers [65]: Nonsignificant reduction if *R* < 0.5, a slight reduction when *R* ≥ 0.5 and < 1, a reduction that was significant when *R* ≥ 1 and < 3, and a reduction was strong if *R* ≥ 3. Experiments were performed in triplicate.

#### 2.4.9. Migration Tests

The specific migration test conditions established by European Normative EC 13130-1:2004 were followed to determine the migration of ZnONPs from the electrospun PHBV-based biopapers. This process was performed by full immersion of the PHBV biopapers containing OEO and ZnONPs (size: 0.5 dm^2^ and weighted accurately) in two food simulants that were sealed in clean wide-mouth jars. These simulant systems consisted of an ethanol solution in water (83.33 mL at 10 vol% at 40 °C for 10 days) and acetic acid in water (83.33 mL at 3% wt/vol at 40 °C for 10 days). The blanks were food simulants without the biopaper samples filled into sealed jars and stored under the same conditions. After the incubation period, that is, 10 days, the biopapers containing OEO and ZnONPs were removed whereas the ethanol and acetic acid simulants were evaporated on an electric hot plate, digested with 1.2 mL of 70% HNO_3_, and resuspended in 12 mL of an ultrapure water vial with 1.2 mL of 70% HNO_3_ vol/vol. The samples were then introduced for metal quantification by Inductively Coupled Plasma-Optical Emission Spectrometry (ICP-OES) using Spectrometer ICPE-9000 (Shimadzu^®^, Kioto, Japan) operating in the wavelength range from 167 nm to 800 nm, and equipped with torch axial or radial configuration, an ultrasonic nebulizer for higher sensitivity, and a charge coupled device (CCD) detector. The wavelength range was set from 167 nm to 800 nm. The instrumental parameters employed for ICP-OES analysis consisted of a nebulizer gas flow (0.70 L/min Ar), an auxiliary gas flow (0.60 L/min Ar), plasma (10 L/min Ar), Ar Gas P (478.66 kPa), and ICP radio frequency (RF) power (1.20 kW), whereas the direction was axial, the rotation speed was 20 rpm, the CCD temperature was −15 °C, and the vacuum level was 6.9 Pa. The linearity of the calibration curve was considered acceptable by achieving a correlation coefficient *R*^2^ > 0.999. All results were blank subtracted and the specific migration tests were performed in triplicate.

### 2.5. Statistical Analysis

The results were evaluated with a 95% significance level (*p* ≤ 0.05) by the analysis of variance (ANOVA). To identify significant differences among the samples, a multiple comparison test (Tukey) was followed using the software OriginPro8 (OriginLab Corporation, Northampton, MA, USA).

## 3. Results and Discussion

### 3.1. Characterization of the Antimicrobial Agents

**Figure 1:**
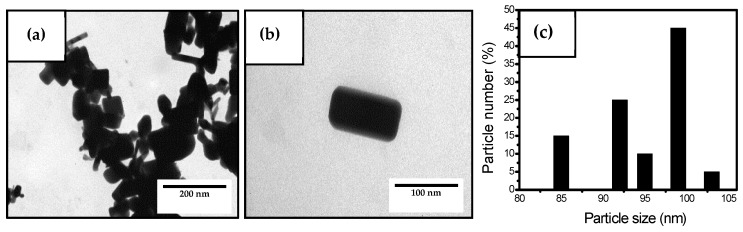
(**a**,**b**) Transmission electron microscopy (TEM) micrographs of zinc oxide nanoparticles (ZnONPs) showing scale markers of 200 nm and 100 nm, respectively. (**c**) Histogram of particle sizes.

TEM was conducted to evaluate the morphology of ZnONPs and their micrographs are presented in Figure 1. In Figure 1a, one can observe the TEM micrograph of the ZnONPs powder, which showed aggregates of cubic-like and rectangular particles with sizes below 100 nm. Figure 1b shows the surface of a single ZnONP, which have a surface of approximately 80 × 20 nm^2^. As observed in the particle histogram shown in Figure 1c, most of the ZnONPs presented sizes between 85 nm and 105 nm, which have an average size of ~98 nm. This morphology is similar to that reported by Naphade and Jog [66] and Ogunyemi et al. [67] who described nanoparticles with elongated and cubic morphologies with lengths from 40 nm to 100 nm and diameters of ca. 50 nm.

Table 1 shows the MIC and MBC values of the neat OEO or ZnONPs against the strains of *S. aureus* and *E. coli*, selected as representative G+ and G− foodborne bacteria, respectively. OEO presents a good antibacterial effect against both bacterial strains, which achieve identical MIC and MBC values, that is, 0.625 μL/mL, against *E. coli*, and 0.312 μL/mL, against *S. aureus* [57]. The similar MIC and MBC values obtained by natural compounds have been described to inhibit the microbial growth and eliminate 99.9% of the microorganisms [68]. In particular, the antimicrobial activity of OEO has been related to its high content in carvacrol and thymol [15]. Further details about the antimicrobial properties of OEO can be found in our previous research works [5,57]. Alternatively, it can be observed that ZnONPs presented the same values of MIC and MBC for both *E. coli* and *S. aureus* bacteria. In particular, the inhibition of the two strains was attained at 0.156 μg/mL. In this regard, Salah et al. [69] reported a MIC value for ZnONPs of 2 μg/mL against *E. coli* and *B. subtilis* bacteria and the *S. cerevisiae* yeast. The differences observed in the antimicrobial activity of the nanoparticles can be related to their morphology and synthesis process. For instance, several authors have indicated that, at high concentrations, aggregates of nanoparticles may form precipitates that decrease the ZnONPs’ antimicrobial activity [35,69]. It is also worthy to mention that the antimicrobial activity of ZnONPs is attributed to the release of ions Zn^2+^, which penetrate the cellular wall of bacteria or adhere to the cell by an electrostatic interaction. This phenomenon leads to the generation of reactive oxygen species (ROS), such as O^2−^ and OH^−^ radicals, on the surface of particles that cause a dysfunction of the membrane and, therefore, the destruction of the bacterial cells [70,71]. Thus, it was confirmed that ZnONPs present a broad spectrum of inhibition for both G+ and G− bacteria [26].

### 3.2. Optimization of the Electrospun PHBV/ZnONPs Biopapers

**Figure 2:**
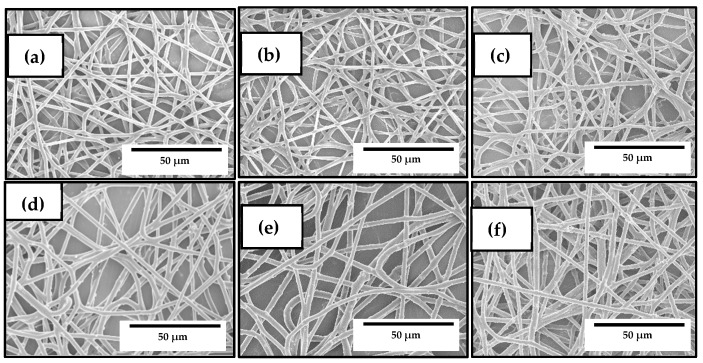
Scanning electron microscopy (SEM) micrographs of the electrospun fibers of poly(3-hydroxybutyrate-*co*-3-hydroxyvalerate) (PHBV): (**a**) Neat PHBV; (**b**) PHBV containing 10 wt% oregano essential oil (OEO); (**c**) PHBV containing 1 wt% zinc oxide nanoparticles (ZnONPs); (**d**) PHBV containing 3 wt% ZnONPs; (**e**) PHBV containing 6 wt% ZnONPs; (**f**) PHBV containing 10 wt% ZnONPs. Scale markers of 50 μm in all cases.

The morphology and the antimicrobial properties of the electrospun PHBV fibers and biopapers containing ZnONPs were also analyzed by SEM and TEM in order to select the optimal content of nanoparticles in the biopolymer. Figure 2 shows the SEM micrographs of the electrospun PHBV fibers. In particular, Figure 2a shows the SEM images of the neat PHBV fibers, whereas Figure 2b includes the PHBV fibers containing 10 wt% OEO, which were also included in the study for comparison. In both cases, the PHBV fibers presented a mean diameter of approximately 0.8 μm, which is agreement with our previous works reporting the morphology of electrospun PHBV fibers [57,58]. Figure 2c–f correspond to the PHBV fibers, without OEO, containing ZnONPs at 1 wt%, 3 wt%, 6 wt%, and 10 wt%. It can be seen that, in all cases, the mean diameters of the fibers were in the 0.88–0.97 μm range. The electrospun PHBV fibers presented a smooth surface morphology without beads. No further changes were observed in the fibers’ morphology after the ZnONPs incorporation with the exception that some nanoparticles were also observed on the fibers surface, which indicates that part of ZnONPs were not incorporated into the PHBV matrix.

**Figure 3:**
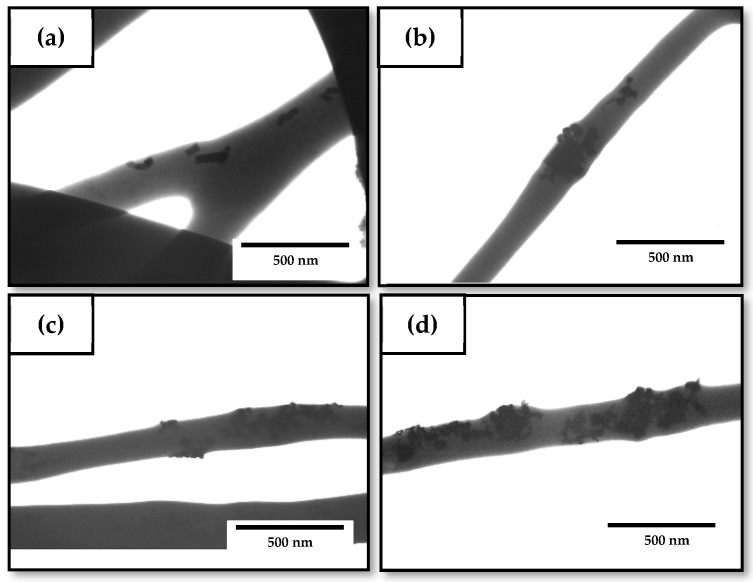
Transmission electron microscopy (TEM) micrographs of the electrospun fibers of poly(3-hydroxybutyrate-*co*-3-hydroxyvalerate) (PHBV) containing zinc oxide nanoparticles (ZnONPs) at: (**a**) 1 wt%; (**b**) 3 wt%; (**c**) 6 wt%; (**d**) 10 wt%. Scale markers of 500 nm in all cases.

Figure 3 shows the TEM micrographs of the nanocomposite fibers to evaluate the dispersion of the nanoparticles within the fibers. In Figure 3a, which corresponds to the fibers containing 1 wt% of ZnONPs, it can be seen that the nanoparticles were efficiently encapsulated in the PHBV fibers during the electrospinning process even though they were randomly distributed within the biopolymer matrix. A similar morphology was attained for the electrospun fibers with a ZnONPs content of 3 wt%, shown in Figure 3b. In general, the nanoparticles showed a relatively good dispersion within the PHBV matrix. However, it can be observed that, as the nanofillers content increased, the ZnONPs tended to form agglomerates. Therefore, the samples containing 6 wt% and 10 wt% ZnONPs (Figure 3c,d, respectively) showed fiber regions with agglomerated ZnONPs. This effect was particularly noticeable for the electrospun PHBV fibers filled with the 10 wt% ZnONPs. This morphological observation supports the previously described thickening observed by SEM for the PHBV fibers attained for the highest ZnONPs loadings.

**Figure 4:**
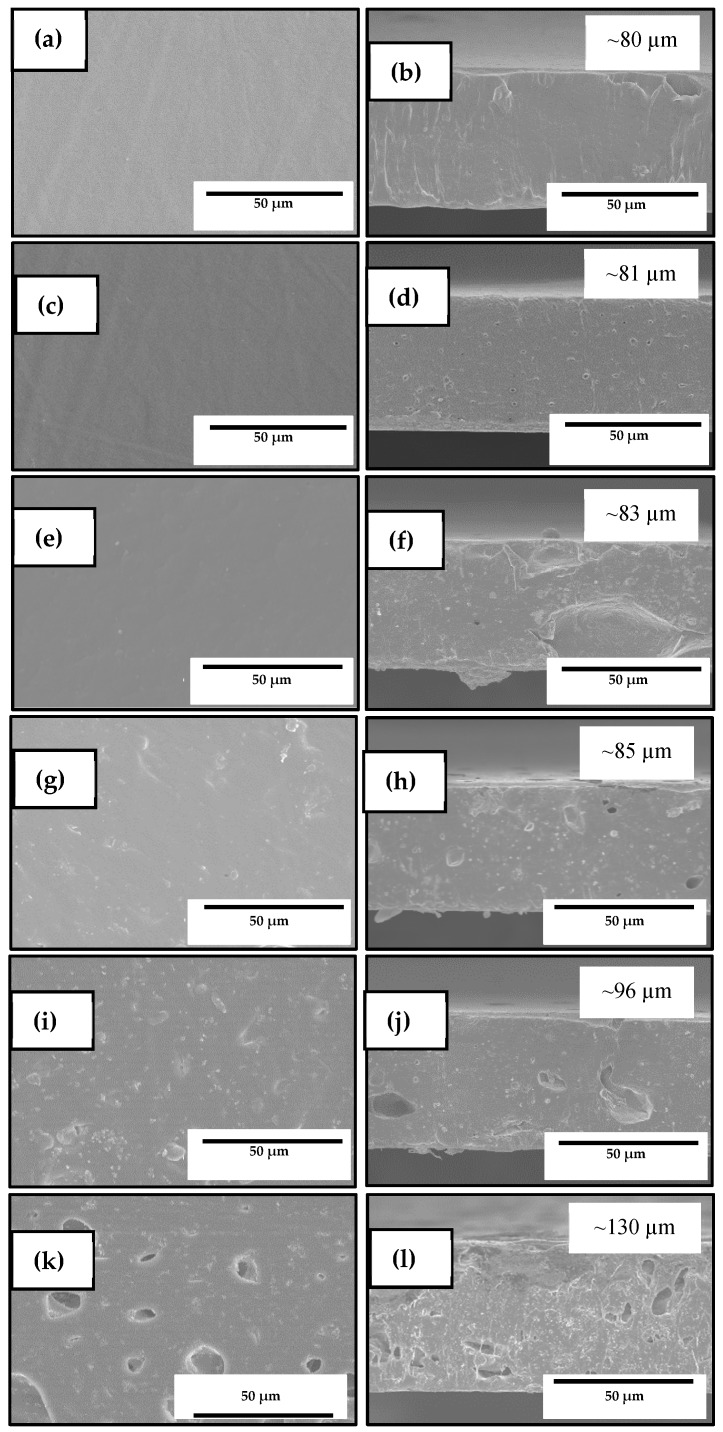
Scanning electron microscopy (SEM) micrographs in a top view (left) and a cross-section (right) of the electrospun biopapers of poly(3-hydroxybutyrate-*co*-3-hydroxyvalerate) (PHBV): (**a**,**b**) Neat PHBV; (**c**,**d**) PHBV containing 10 wt% oregano essential oil (OEO); (**e**,**f**) PHBV containing 1 wt% zinc oxide nanoparticles (ZnONPs); (**g**,**h**) PHBV containing 3 wt% ZnONPs; (**i**,**j**) PHBV containing 6 wt% ZnONPs; (**k**,**l**) PHBV containing 10 wt% ZnONPs. Scale markers of 50 μm in all cases.

The resultant electrospun mats were annealed at 125 °C in order to produce continuous films composed of fibers, which are called biopapers. Figure 4 shows the SEM images of the electrospun materials after the thermal post-treatment at 125 °C in both their cross-section and top views. The biopapers of neat PHBV, presented in Figure 4a,b and of the PHBV containing 10 wt% OEO, presented in Figure 4c,d, showed a similar thickness of ~80 μm. The neat PHBV biopaper exhibited a homogeneous and continuous structure, which is similar to the morphologies reported recently by Melendez-Rodriguez et al. [61], even though, in the previous study, the films showed a higher porosity due to the presence of high loadings of eugenol that plasticized the PHBV matrix, which was evaporated/migrated during the annealing process. Similarly, the OEO-containing PHVB biopapers presented some small pores in their cross section due to the evaporation of the volatiles during the thermal post-treatment. The top view and cross section of the PHBV biopapers containing 1 wt% ZnONPs are gathered in Figure 4e,f, where it can be observed that the biopaper surface was also homogenous. Similar morphologies can be seen in Figure 4g,h for the PHBV biopapers filled with 3 wt% ZnONPs. However, in the case of the biopapers containing 6 wt%, shown in Figure 4i,j and 10 wt% ZnONPs, in Figure 4k,l, the presence of nanoparticles altered their surface, which affected the film homogeneity and generated cracks. The presence of ZnONPs also increased significantly the biopaper thicknesses to ~130 μm, in all the biopaper samples, which can be related to confinement restrictions of the fibers’ reorganization by the nanoparticles’ presence during annealing.

**Figure 5:**
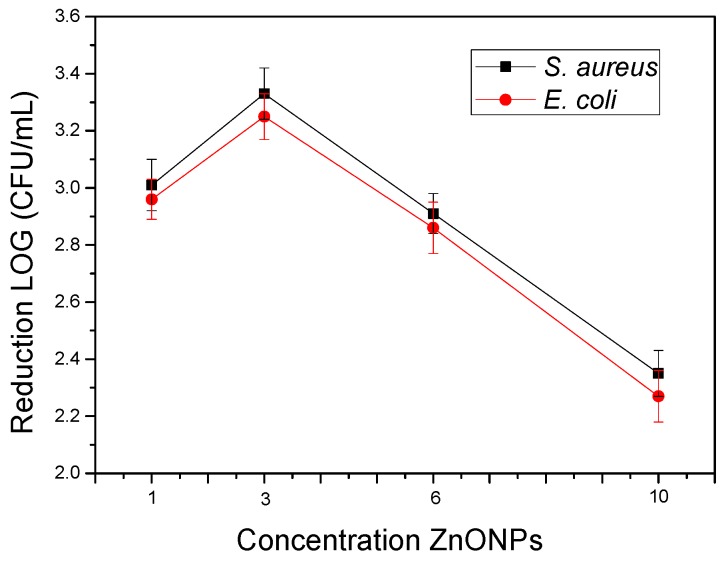
Antimicrobial properties of the electrospun biopapers of poly(3-hydroxybutyrate-*co*-3-hydroxyvalerate) (PHBV) containing different contents of zinc oxide nanoparticles (ZnONPs) against *Staphylococcus aureus* (*S. aureus*) and *Escherichia coli* (*E. coli*) in an open system for 24 h.

Lastly, the antimicrobial properties in an open system of the electrospun PHBV biopapers filled with the different contents of ZnONPs against *S. aureus* and *E. coli* were studied and the results are gathered in Figure 5. Film samples with concentrations of 1 wt% and 3 wt% ZnONPs showed strong inhibition (*R* ≥ 3) against both strains after 24 h. Interestingly, when the concentration of ZnONPs increased, the antimicrobial activity decreased. This effect can be related to the previously mentioned phenomenon of agglomeration, once the viable active surface of ZnONPs was reduced when they formed agglomerates and, therefore, reduced the ion-releasing process and, thus, their effectiveness. As reported earlier, the size, morphology, and specific surface area of ZnONPs can greatly affect the antibacterial properties and, thus, the agglomeration of ZnONPs must be avoided [35]. These results are also in accordance with a previous work of Padmavathy and Vijayaraghavan [72] who determined that the antibacterial activity of ZnO against *E. coli* was stronger when the particle size decreased and the particle dispersion was improved.

Based on the obtained results, 3 wt% was selected as the optimal content of ZnONPs in PHBV. The resultant electrospun biopapers were not only more uniform and homogeneous, but they also offered the highest antibacterial properties at a relatively low content. The here-attained optimal content of ZnONPs in PHBV was two times lower than that previously obtained by Castro-Mayorga et al. [59] who reported a reduction of approximately 3 log CFU/mL for *L. monocytogenes* with PHBV films filled with 6 wt% ZnONPs. The differences observed can be related to methods employed for the incorporation of the ZnONPs such as direct melt-mixing, melt-mixing of pre-incorporated ZnONPs into PHBV, and coating of annealed electrospun fiber mats over compression-molded PHBV films as well as the morphology and crystal-type of the nanoparticles.

### 3.3. Development of the Electrospun Hybrid PHBV Biopapers

**Figure 6:**
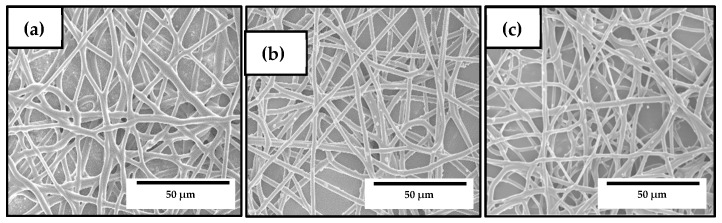
Scanning electron microscopy (SEM) micrographs of the electrospun fibers of poly(3-hydroxybutyrate-*co*-3-hydroxyvalerate) (PHBV) containing different amounts of oregano essential oil (OEO) and zinc oxide nanoparticles (ZnONPs): (**a**) PHBV + 7.5 wt% OEO + 0.75 wt% ZnONPs; (**b**) PHBV + 5 wt% OEO + 1.5 wt% ZnONPs; (**c**) PHBV + 2.5 wt% OEO + 2.25 wt% ZnONPs. Scale markers of 50 μm in all cases.

The PHBV fibers containing the mixtures of OEO and ZnONPs, known as the hybrid fibers, were developed and compared to the previously prepared electrospun materials based on either neat PHBV or PHBV with 10 wt% OEO or 3 wt% ZnONPs. Figure 6 shows the SEM micrographs of the electrospun hybrid PHBV fibers. In Figure 6a, one can observe the morphology of the PHBV fibers containing 7.5 wt% OEO + 0.75 wt% ZnONPs. The mean diameter was 0.90 ± 0.28 μm and the fibers were smooth and also free of beaded regions. Similar morphologies were attained for the PHBV fibers containing 5 wt% OEO + 1.5 wt% ZnONPs and 2.5 wt% OEO + 2.25 wt% ZnONPs, respectively shown in Figure 6b,c. The fibers’ diameters, however, slightly increased (*p* ≥ 0.005) with the increase of the ZnONPs content and some nanoparticles were also placed outside the fibers. In particular, the mean diameters were 0.92 ± 0.32 μm and 0.94 ± 0.41 μm, for the PHBV fibers containing 5 wt% OEO + 1.5 wt% ZnONPs and 2.5 wt% OEO + 2.25 wt% ZnONPs, respectively. A similar effect was observed by Mousavi et al. [73] where the addition of cerium dioxide (CeO_2_)/dendrimer nanoparticles significantly increased the diameter of electrospun pullulan/poly(vinyl alcohol) (PVA)/poly(acrylic acid) (PAA) fibers and it also induced surface roughness. This effect was ascribed to the high filler concentration, which promoted porosity and fiber thickening. In general, all the PHBV-based fibers presented a uniform and smooth surface, showing no surface or structural defects, which indicates that the addition of mixtures of OEO and ZnONPs positively did not alter fiber formation during electrospinning.

**Figure 7:**
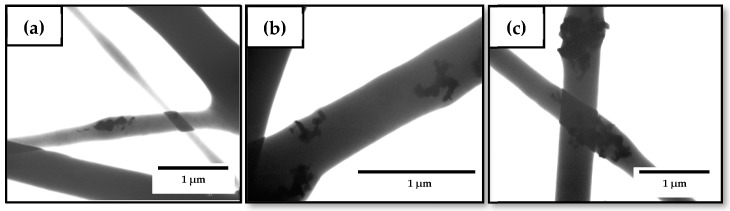
Transmission electron microscopy (TEM) micrographs of the electrospun fibers of poly(3-hydroxybutyrate-*co*-3-hydroxyvalerate) (PHBV) containing different amounts of oregano essential oil (OEO) and zinc oxide nanoparticles (ZnONPs): (**a**) PHBV + 7.5 wt% OEO + 0.75 wt% ZnONPs; (**b**) PHBV + 5 wt% OEO + 1.5 wt% ZnONPs; (**c**) PHBV + 2.5 wt% OEO + 2.25 wt% ZnONPs. Scale markers of 1 μm in all cases.

The morphology of the electrospun hybrid PHBV fibers was also analyzed by TEM to observe the effect of OEO on the dispersion of ZnONPs in the biopolymer matrix. Figure 7 gathers the TEM micrographs of the electrospun fibers. One can observe in Figure 7a,b, corresponding to the PHBV fibers containing 7.5 wt% OEO + 0.75 wt% ZnONPs and 5 wt% OEO + 1.5 wt% ZnONPs, respectively, that the nanoparticles were well dispersed along the biopolymer even though they also tended to agglomerate in certain regions. Agglomeration was more intense in the case of the PHBV fibers containing 2.5 wt% OEO + 2.5 wt% ZnONPs, shown in Figure 7c, suggesting that larger aggregate structures were formed as the nanoparticles’ concentration increased. This phenomenon is in agreement with the results reported by Cherpinski et al. [74] who incorporated palladium nanoparticles (PdNPs) into electrospun fibers of PHB. Nanoparticle agglomeration can be ascribed to the large surface area of the nanoparticles and the electrostatic forces among them [75]. The hybrid fibers showed lower agglomerates of the nanoparticles when compared with the ZnONPs-containing PHBV fibers without OEO, as seen in Figure 3, which suggests that the presence of the oil favored the dispersion of the nanoparticles. This effect can be ascribed to a potential plasticization of the PHBV matrix that could facilitate cluster breakup and separation of nanoparticles during electrospinning.

**Figure 8:**
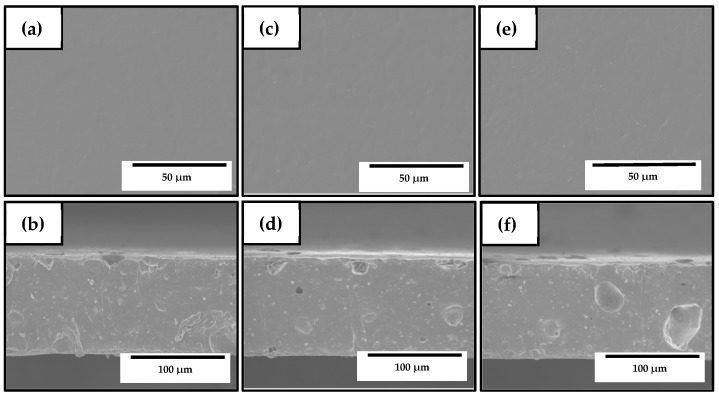
Scanning electron microscopy (SEM) micrographs in top view (top) and cross-section (bottom) of the electrospun biopapers of poly(3-hydroxybutyrate-*co*-3-hydroxyvalerate) (PHBV) containing different amounts of oregano essential oil (OEO) and zinc oxide nanoparticles (ZnONPs): (**a**,**b**) PHBV + 7.5 wt% OEO + 0.75 wt% ZnONPs; (**c**,**d**) PHBV + 5 wt% OEO + 1.5 wt% ZnONPs; (**e**,**f**) PHBV + 2.5 wt% OEO + 2.25 wt% ZnONPs. Scale markers of 50 μm and 100 μm, respectively.

Figure 8 shows the SEM images of the electrospun materials after annealing at 125 °C in their cross-section and top views. On the top views of the samples included in Figure 8a,c,e, it can be observed that all the biopapers exhibited a homogeneous surface without cracks and/or pores, similar to those SEM images shown in Figure 4 for electrospun PHBV filled with ZnONPs. However, all the biopapers also showed several pores in the cross-section, which can be related to the partial evaporation of the oily particles derived from OEO enclosed in the PHBV matrix during the thermal post-treatment as well as the presence of ZnONP agglomerates. Similar voids, though smaller, were recently observed in electrospun PHBV filled with silica microparticles containing eugenol prepared by Melendez-Rodriguez et al. [58]. The pores were significantly bigger for the PHBV biopaper containing 2.5 wt% OEO + 2.25 wt% ZnONPs, shown in Figure 8f, in comparison with the cross-sections of the biopaper samples of PHBV containing 7.5 wt% OEO + 0.75 wt% ZnONPs and 5 wt% OEO + 1.5 wt% ZnONPs, respectively, which are presented in Figure 8b,d. This observation confirms that the formation of large voids was mainly ascribed to the nanoparticle agglomerates, as previously observed in Figure 7c. On the other hand, Ejaz et al. [46] reported a significant increase in the thickness of gelatin type B films obtained by casting when ZnONPs were incorporated in combination with CEO. Likewise, Castro-Mayorga et al. [59] obtained thicker films after the incorporation of ZnONPs, which was also influenced by the amount and shape of nanoparticles.

### 3.4. Optical Properties of the Electrospun Hybrid PHBV Biopapers

**Figure 9:**
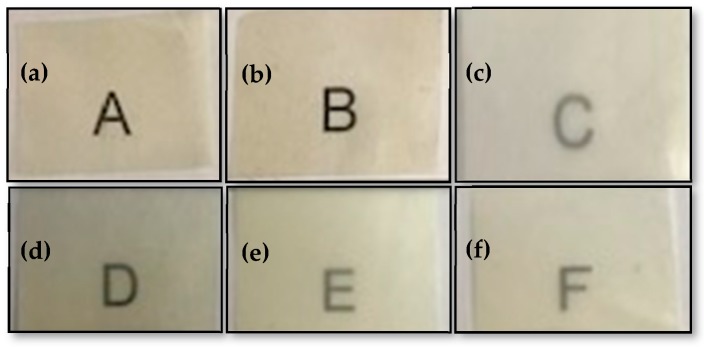
Visual aspect of the electrospun biopapers of poly(3-hydroxybutyrate-*co*-3-hydroxyvalerate) (PHBV): (**a**) Neat PHBV, (**b**) PHBV + 10 wt% oregano essential oil (OEO), (**c**) PHBV + 3 wt% zinc oxide nanoparticles (ZnONPs), (**d**) PHBV + 7.5 wt% OEO + 0.75 wt% ZnONPs, (**e**) PHBV + 5 wt% OEO + 1.5 wt% ZnO-NPs, and (**f**) PHBV + 2.5 wt% OEO + 2.25 wt% ZnONPs.

Figure 9 shows the visual aspect of the electrospun PHBV biopapers for evaluating their contact transparency. The effect of the combined addition of OEO and ZnONPs on the color coordinates (L*, a*, b*) and the values of Δ*E*, *T*, and *O* of the electrospun PHBV biopapers are shown in Table 2. The optical properties of the neat PHBV biopaper and the PHBV biopapers with either 10 wt% OEO or 3 wt% ZnONPs were also included for comparison purposes. It can be observed that all PHBV-based biopapers presented contact transparency but the presence of both OEO and ZnONPs reduced brightness and increased opacity, which was measured by the L* and *O* values, respectively. The biopapers develop a low-intense yellow color when OEO was incorporated, which was confirmed by the increase in the *b* * coordinate. Transparency was noticeably reduced with the ZnONPs content even though the effect of the nanoparticles on the color change was relatively low. On the opposite side, all the OEO-containing biopapers resulted in samples in which an observer can notice different colors (ΔE* ≥ 5). For instance, the Δ*E* value of the PHBV biopapers only containing OEO was 8.36, whereas this value for the biopapers only containing ZnONPs was 1.83. Therefore, the biopapers containing OEO and ZnONPs mixtures presented intermediate Δ*E* values and the color was more affected by the OEO content. Therefore, among the hybrid PHBV biopapers, the highest color change was observed for the PHBV film containing 7.5 wt% OEO + 0.75 wt% ZnONPs, that is, 7.64.

Therefore, the presence of ZnONPs decreased transparency, showing *T* values from 3.13, for the neat PHBV biopaper, to 9.60, for the PHBV biopaper filled with 3 wt% ZnONPs. Similarly, it increased opacity, which presented *O* values from 0.016 to 0.052. Thus, the addition of ZnONPs did not strongly affect the visual color of the samples, but the measurements of these parameters showed statistically significant differences (*p* < 0.05). Moreover, it showed a significantly reduction in the transmission of visible and ultraviolet (UV) light of the electrospun PHBV biopapers by changing the scattering of light. This capacity to block UV light is intrinsically attributed to ZnO, which additionally shows photocatalytic activity [26,76] and can influence the optical properties of films. In this regard, Wang et al. [77] determined that the large ZnO contents in carboxymethyl/chitosan films increased the UV absorption due to the dispersion of light generated by the high crystallinity of this filler. In this regard, films with UV block properties can be of great interest in food packaging for application in photosensitive products.

### 3.5. Thermal Stability of the Electrospun Hybrid PHBV Biopapers

**Figure 10:**
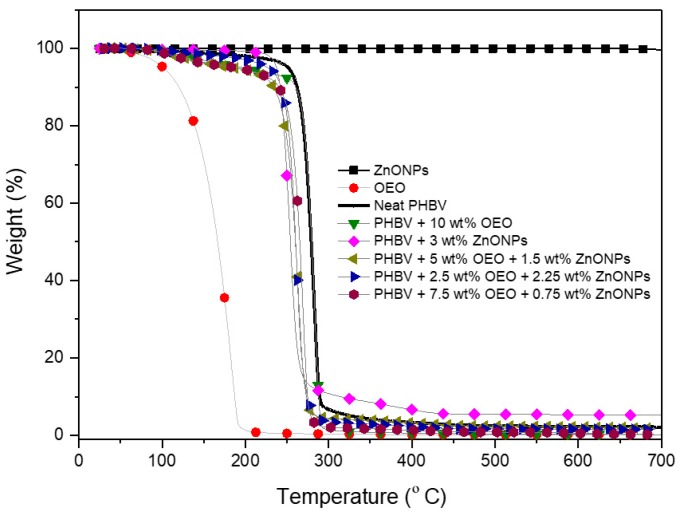
Evolution of weight (%) as a function of temperature of the zinc oxide nanoparticles (ZnONPs), oregano essential oil (OEO), and electrospun biopapers of poly(3-hydroxybutyrate-*co*-3-hydroxyvalerate) (PHBV) containing OEO and ZnONPs.

The weight loss curves of OEO, ZnONPs, and of the electrospun PHBV biopapers obtained by TGA are gathered in Figure 10. The values of the onset degradation temperature, that is, the temperature at 5% weight loss (*T_5%_*), degradation temperature (*T_deg_*), and residual mass at 700 °C are summarized in Table 3. One can observe that OEO presented a low thermal stability, which shows values of *T_5%_* and *T_deg_* of 101.5 °C and 178.4 °C, respectively, with a respective weight loss of 74.16% at *T_deg_*, corresponding to the volatilization and/or degradation of low-molecular weight (*M_W_*) volatile compounds present in the OEO (e.g., carvacrol, thymol, and pinene) [57]. On the opposite end, it can be observed that ZnONPs were thermally stable in the whole range of temperatures tested.

The neat PHBV biopaper was thermally stable up to 251.5 °C, which shows a *T_deg_* value of 278.7 °C (47.74 wt%) and a residual mass of 2.10 wt%. These thermal values are relatively similar to those reported for PHBV materials in previous research works [78,79,80], where the thermal decomposition reaction of the biopolymer chain occurred sharply in one single and sharp step from approximately 270 °C to 280 °C. The presence of OEO considerably reduced the onset of degradation of PHBV, which shows a *T_5%_* value of 197.5 °C. The ZnONPs-containing PHBV biopaper showed a higher *T_5%_* value but lower value of *T_deg_* than the neat PHBV biopaper, which could be attributed to the thermal conductivity and catalytic properties of the ZnONPs. This effect on the thermal properties is similar to that reported by Castro-Mayorga et al. [59] for electrospun PHBV films containing 6 wt% ZnONPs. All the PHBV biopapers containing the OEO and ZnONPs mixtures showed a similar thermal decomposition process with a unique degradation stage that started around ∼260 °C. Although the values of *T_deg_* were reduced, the loss of mass at *T_deg_* was smaller compared with those of neat PHBV and OEO-containing PHBV biopapers. This may indicate that ZnONPs delayed the thermal degradation process of PHBV. In this regard, the incorporation of ZnONPs can improve the thermal resistance of polymer materials due to the barrier effect of the nanoparticles that could hinder the transport of decomposition products from the bulk biopolymer matrix. Likewise, the high thermal conductivity of ZnO can also help heat dissipation within the composite, which results in enhanced thermal stability [81]. Furthermore, all the PHBV biopapers showed a residual mass in the range of 2–5%, which increased slightly with the ZnONPs’ content.

### 3.6. Mechanical Properties of the Electrospun Hybrid PHBV Biopapers

Tensile modulus (E), tensile strength at yield ($\sigma_{y}$), and elongation at break ($\varepsilon_{b}$) were calculated from the stress–strain curves, estimated from the force (*F*) vs. distance (*d*) data. The mechanical properties of the electrospun PHBV-based biopapers are shown in Table 4. The biopaper made of neat PHBV presented an *E* value of 1125 MPa, a *σ_y_* value of 12.6 MPa, and a *ε_b_* value of 1.71%. These results are in the same range than those reported by Melendez-Rodriguez et al. [58] for electrospun films of neat PHBV, where *E* was 1252 MPa, *σ_y_* was 18.1 MPa, and *ε_b_* was 2.4%. The incorporation of OEO reduced the *E* value to 814 MPa, which can be ascribed to a plasticizing effect of the oil molecules on the biopolymer matrix, which reduced the intermolecular forces and increased the mobility of the PHBV chains [82]. Thus, it increased both the *σ_y_* and *ε_b_* to values of 18 MPa and 4.53%, respectively, and also toughness from 0.124 mJ/mm^3^ to 0.560 mJ/mm^3^. A similar improvement in ductility was reported by Melendez-Rodriguez et al. [58] after the introduction of eugenol in electrospun PHBV films. In relation to ZnONPs, one can observe that the nanoparticles induced a reinforcement on PHBV so that the values of *E* and *σ_y_* increased in these film samples to 1286 MPa and 17.1 MPa and, as expected, it resulted in a decrease in ductility showing a value of *ε_b_* of 1.26%. Similarly, Díez-Pascual et al. [81] reported a mechanical strength improvement with the addition of ZnONPs in the range of 1–5 wt% in PHB, which shows an increase in the *E* and *σ_y_* values of up to 43% and 32%, respectively. The reinforcement attained can be influenced by different factors such as load dispersion, the degree of crystallinity of the polymer, and the interfacial adhesion of the nanoparticles to the biopolymer matrix. The electrospun PHBV biopapers containing the OEO and ZnONPs mixtures presented a similar mechanical resistance, which showed *E* values in the 700–900 MPa range. This is very similar to that of the OEO-containing PHBV biopaper, whereas the values of *σ_y_* remained in the range of 14–15 MPa. As the content of OEO in the biopaper increased, the flexibility and toughness of the samples improved. Thus, the electrospun PHBV biopapers containing 7.5 wt% OEO + 0.75 wt% ZnONPs showed the lowest *E* value, that is, 778 MPa, but also the highest *ε_b_* value, that is, approximately 5%. In this regard, Ejaz et al. [46] reported a decrease in the *σ_y_* values and an increase in the *ε_b_* values for bovine skin gelatin films filled with 2 wt% ZnONPs in combination with cinnamon oil. Chun et al. [83] also found that the addition of silver nanoparticles (AgNPs) and ZnONPs decreased the rigidity and tensile strength of PLA films. This effect was ascribed to a reduction of the biopolymer’s *M_W_*, which improved the mobility of the PLA chains by a chain-scission process during processing.

### 3.7. Crystallinity of the Electrospun Hybrid PHBV Biopapers

**Figure 11:**
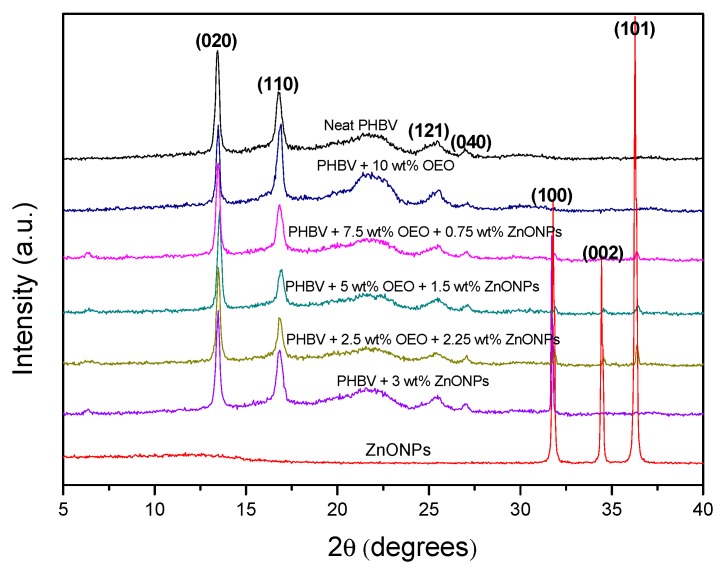
Diffractograms of the zinc oxide nanoparticles (ZnONPs) and electrospun biopapers of poly(3-hydroxybutyrate-*co*-3-hydroxyvalerate) (PHBV) containing oregano essential oil (OEO) and ZnONPs.

Wide Angle X-ray Diffraction (WAXD) experiments were conducted on the electrospun biopapers of PHBV to ascertain their crystallinity. The diffractograms are plotted in Figure 11. Most of the characteristic peaks at 2*θ* of PHBV were clearly detected for the neat PHBV diffractogram, that is, 13.4°, 16.9°, 25.5°, and 27.1°. According to the literature [84], these peaks correspond to the (020), (110), (121), and (040) lattice planes of the orthorhombic unit cells of PHB. The PHB crystal lattice is characteristic for the PHBV with HV contents below 37% [85]. This is the case of the present PHBV. There were no changes in position of the PHBV diffraction peaks with the addition of OEO or ZnONPs, which suggests that the crystalline structure of PHBV was not altered. One can also observe that ZnONPs presented three main peaks in the studied range, corresponding to the (100), (002), and (101) reflections. The presence of these peaks in the diffractograms of the ZnONPs-containing PHBV sample confirmed the presence of the nanoparticles in the biopaper. The low relative intensity of these peaks in the samples can be attributed to a dilution effect.

### 3.8. Antimicrobial Activity of the Electrospun Hybrid PHBV Biopapers

**Figure 12:**
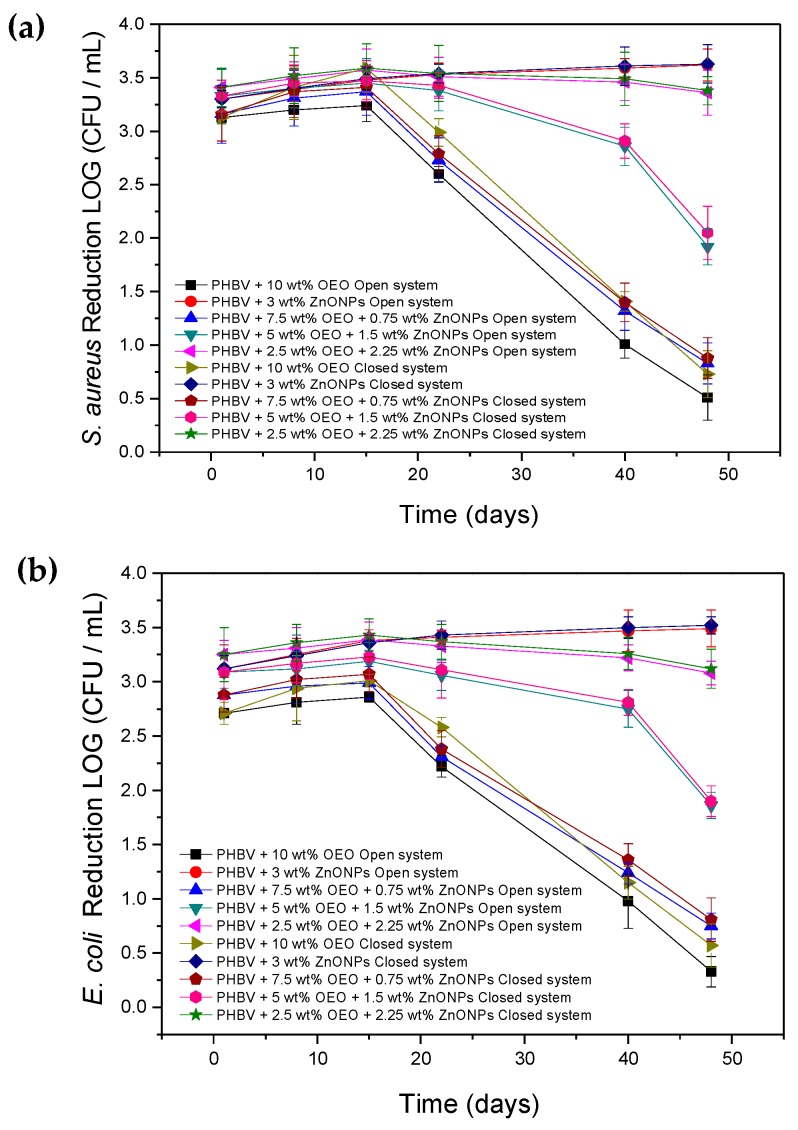
Antimicrobial activity of the electrospun biopapers of poly(3-hydroxybutyrate-*co*-3-hydroxyvalerate) (PHBV) containing oregano essential oil (OEO) and zinc oxide nanoparticles (ZnONPs) against (**a**) *S. aureus* and (**b**) *E. coli* in the open and closed systems for 48 days.

Figure 12 gathers the results of antimicrobial activity in an open and closed system, respectively, for 48 days, of the electrospun PHBV biopapers containing different OEO and ZnONP mixtures against the *S. aureus* and *E. coli* strains. The antimicrobial properties of the PHBV biopapers containing OEO 10 wt% and ZnONPs 3 wt% are also included for comparison. It can be observed that the OEO-containing PHBV biopaper presented a strong reduction (*R* ≥ 3) for *S. aureus* and a significant reduction (*R* ≥ 1 and < 3) of *E. coli* for 15 days in both systems, which is slightly higher in the closed system. This slight difference between the two tested systems can be attributed to the release and accumulation of volatile OEO compounds into the closed headspace, which contributed to a greater inhibition of bacterial growth. From day 22, however, the inhibition of the PHBV biopaper for both strains decreased significantly due to the complete release of the active volatile compounds, which resulted in a decrease of the active properties of the biopapers. In the case of the PHBV biopapers containing ZnONPs 3 wt%, the reduction achieved was slightly lower for both bacteria and systems in comparison with that achieved for the OEO-containing PHBV biopapers during the first 15 days. However, it was still strong (*R* ≥ 3) and, interestingly, it also improved during the 48 days of the assay. The strong activity of ZnONPs can be attributed to different mechanisms of action in the presence of moisture, such as the release of Zn^2+^ ions, reactive oxygen species (e.g., superoxide, hydroxyl, and hydrogen peroxide radicals) that are able to penetrate the bacterial wall and cause irreversible damage to the bacterial cellular structure [86]. Therefore, the antibacterial activity can be improved with time as the nanoparticles remained active by contact and due to the high humidity of both systems that favored their release and accumulation on the biopaper surface. It is also worthy to mention that, in all cases, the results obtained showed that the G+ bacteria are more sensitive than G− ones to both OEO and ZnONPs. As reported by other authors, the cell wall structure of *E. coli* is composed mainly of lipopolysaccharides and a thin layer of peptidoglycan that hinders the penetration of negatively charged reactive oxygen species formed by the presence of ZnONPs [87].

The electrospun hybrid PHBV biopapers, that is, the fiber-based film samples containing the different mixtures of OEO and ZnONPs, also showed a high antimicrobial activity against both bacterial strains in the two systems. During the first 15 days, that is, in the short term, the PHBV biopaper containing 2.5 wt% OEO + 2.25 wt% ZnONPs showed the highest reduction values among the materials tested. The fact that the antimicrobial activity of the hybrid biopaper at this particular composition was even slightly higher than both biopapers based on each antimicrobial agent reveals the synergism attained in the combination of OEO and ZnONPs. This observation suggests that the release of OEO from the PHBV matrix was improved due to the presence of the nanoparticles and/or the antimicrobial effectiveness of the inorganic nanoparticles was enhanced. In the latter case, previous studies have demonstrated that vegetal extract containing polyphenols, such as the tannins, glycosides, and flavonoids, could act as reducing and capping agents [88]. Therefore, the reaction between nanoparticles and the polyphenolic compounds carvacrol and thymol, which are the major constituents of OEO, can potentially improve the antimicrobial activity of ZnONPs. Furthermore, the PHBV biopapers containing 5 wt% OEO + 1.5 wt% ZnONPs and 7.5 wt% OEO + 0.75 wt% ZnONPs presented intermediate values of reduction but are still strong (*R* ≥ 3). Then, both biopapers showed a performance reduction in the antimicrobial activity with time similar to that observed for the OEO-containing PHBV biopaper. On day 48, this reduction was significant (*R* ≥ 1 < 3) for the PHBV biopapers containing 5 wt% OEO + 1.5 wt% ZnONPs and slight (*R* ≥ 0.5 and < 1) in the case of the PHBV biopaper containing 7.5 wt% OEO + 0.75 wt% ZnONPs. The time evolution in the antimicrobial activity of the PHBV biopaper containing 2.5 wt% OEO + 2.25 wt% ZnONPs was relatively similar to that observed for the PHBV biopaper filled with 3 wt% ZnONPs, even in the conditions of the open system. Therefore, the strong reduction (*R* ≥ 3) observed in the long term of these electrospun hybrid biopapers can be mainly attributed to the ZnONPs presence, which successfully managed to maintain the antimicrobial activity against the two bacterial strains in both systems.

The long-term antimicrobial performance of different nanoparticles has been previously employed to develop different active films. For instance, Emamifar et al. [28] studied the antimicrobial effect of low-density polyethylene (LDPE) films containing TiO_2_ 95 wt% + AgNPs 5 wt% and ZnONPs. The results showed that the microbial growth rate of *Lactobacillus plantarum* (*L. plantarum*) was significantly reduced for loadings of 1.5–5 wt% and 0.25–1 wt% of TiO_2_ + AgNPs and ZnONPs, respectively, during 112 days of study. This points out that the antimicrobial activity of ZnONPs significantly outperforms that of TiO_2_ + AgNPs. Chu et al. [83] also achieved a significant reduction of 2–3 Log_10_ CFU/mL in the growth of *E. coli* by using PLA films containing AgNPs 0.5 wt% and ZnONPs 3 wt% after 12 h of study. Therefore, the relative low content of nanoparticles used in the here-developed hybrid film, that is, 2.25 wt%, represents a technological achievement to develop antimicrobial performance with short-term and long-term performance. In this regard, Ejaz et al. [46] also determined a strong antimicrobial activity of bovine skin gelatin films containing ZnONPs 2 wt% + CEO 50 wt% against *L. monocytogenes* and *S. typhimurium* tested in vitro for 20 days at a refrigerated storage. This result confirms the potential synergism between EOs and nanoparticles, which were able to penetrate through the bacterial cell membrane to alter the cell structure.

### 3.9. Migration Assessment of the Electrospun Hybrid PHBV Biopapers

The amount of ZnONPs that migrate from the PHBV biopapers were tested in different food simulants. Results showed that a greater amount of zinc was released in the acidic solution than in the alcoholic solution. The amount of zinc migrated from the PHBV-based biopapers increased for the samples with higher concentrations of ZnONPs, which shows values of 1.28 ± 0.58 mg/L (PHBV + 7.5 wt% OEO + 0.75 wt% ZnONPs), 3.91 ± 1.64 mg/L (PHBV + 5 wt% OEO + 1.5 wt% ZnONPs), 6.05 ± 0.81 mg/L (PHBV + 2.5 wt% OEO + 2.25 wt% ZnONPs), and 12.87 ± 2.04 mg/L (PHBV + 3 wt% ZnONPs). This behavior could be caused by the high solubility of ZnONPs in acetic acid that, on the one hand, triggered a higher release of it in the food simulant and, on the other hand, it can promote partially acidolysis of the polymer. The latter phenomenon could be observed with the naked eye in the PHBV + 3 wt% ZnONPs sample in 3% (wt./vol.) acetic acid. Similar results were reported by the European Food Safety Authority (EFSA) [89] where the migration of zinc uncoated and coated with [3-(methacryloxy)propyl] trimethoxysilane (MEMO) into 3% acetic acid reached 17.3 mg/kg, whereas, in 10% ethanol, it reached values up to 80 µg/kg. The amount of zinc released in the alcoholic solution showed values below the limit allowed. In particular, the values obtained were 0.45 ± 0.04 mg/L (PHBV + 7.5 wt% OEO + 0.75 wt% ZnONPs), 0.61 ± 0.1 mg/L (PHBV + 2.5 wt% OEO + 2.25 wt% ZnONPs), and 0.62 ± 0.2 mg/L (PHBV + 3 wt% ZnONPs). In light of the above findings, and, according to the current specific migration limit (SML), 5 mg/kg food or food simulant for soluble ionic zinc was set out by the European Plastics Regulation (EU 2016/1416) [90]. The PHBV biopapers containing 3 wt% ZnONPs and 2.5 wt.% OEO + 2.25. wt% ZnONPs exceed the current SML value in acid aqueous solutions, whereas the PHBV biopapers containing 5 wt% OEO + 1.5 wt% ZnONPs and 7.5 wt% OEO + 0.75 wt% ZnONPs comply with the current regulation. In the ethanol aqueous food simulants, all the tested PHBV biopapers were under the current SML values. This result suggests that the present PHBV-based biopapers can be used as food packaging for alcoholic foods but, for acidic food, biopapers with a concentration above 2.25% ZnONPs are restricted.

## 4. Conclusions

The non-renewable origin of most currently used polymers and their lack of biodegradability is behind their excessive carbon and water footprints and also waste management concerns, which results in an increased research interest in the development of more environmentally compatible and economically sustainable packaging materials. Current strategies to solve these problems within Circular Bioeconomy scenarios include the development of biopolymers obtained from agricultural wastes and food processing by-products, the second generation of feedstock, and the application of nanotechnology and active packaging technologies to tailor their properties. In the case of PHAs, their production by mixed cultures derived from biowaste can also represent an opportunity to reduce the costs of the fermentation and downstream processes by the use of a cost-effective raw material. In this research, hybrid submicron fibers of biowaste derived PHBV containing varying amounts of OEO and ZnONPs were successfully developed by electrospinning. The electrospun mats were turned into actual films of ~130 μm by applying a thermal post-treatment at 125 °C. The resultant PHBV-based biopapers exhibited a homogeneous surface even though they also showed some cracks and/or pores due to the partial volatilization of OEO and the presence of ZnONPs agglomerates. The biopapers showed contact transparency, but they also developed a slightly yellow appearance when the OEO was incorporated and a higher opacity with the increase of ZnONPs content. The dual incorporation of OEO and ZnONPs decreased the tensile modulus but positively increased the ductility and toughness of the biopapers, whereas the crystallinity of PHBV was unaffected. The OEO-containing PHBV biopapers showed a strong inhibition for the first 15 days of storage (short-term inhibition). However, it decreased from day 22 due to the complete release of the volatile compounds. Alternatively, the electrospun PHBV biopapers containing ZnONPs showed a high and slightly increasing antimicrobial activity with time for 48 days (long-term inhibition). The electrospun hybrid PHBV biopapers containing 2.5 wt% OEO + 2.25 wt% ZnONPs attained the highest antimicrobial properties in the short term and also high performance in the long term. Lastly, the migration tests performed on the biopaper samples containing ZnONPs revealed that the nanoparticles tend to easily release in acidic solutions due to the partial solubility of PHBV to this medium, whereas they comply with the current SML values for food simulants based on aqueous ethanol (10%). Although the main antimicrobial activity was related to the presence of ZnONPs, their combination with OEO yielded biopapers with high reduction values using lower contents of the inorganic nanoparticles. Furthermore, the presence of the essential oil can also add additional active functionalities such as the previously reported antioxidant activity to extend shelf-life even further. Therefore, novel antimicrobial biopapers with high potential applications in the food packaging field can be successfully prepared by electrospinning and subsequent annealing of biopolymers incorporating low OEO contents and moderate-to-low ZnONP content. Potential applications of the developed biopapers are foreseen in active food packaging for the protection and preservation of perishable foods.

## Figures and Tables

**Table 1:** Minimum inhibitory concentration (MIC) and minimum bactericidal concentration (MBC) of the neat oregano essential oil (OEO) and zinc oxide nanoparticles (ZnONPs) against *Staphylococcus aureus* (*S. aureus*) and *Escherichia coli* (*E. coli*).

Active Agent	Bacteria	MIC (µL/mL)	MBC (µL/mL)
OEO	*E. coli*	0.625	0.625
OEO	*S. aureus*	0.312	0.312
ZnONPs	*E. coli*	0.156	0.156
ZnONPs	*S. aureus*	0.156	

**Table 2:** Color parameters and transparency characteristics of the electrospun biopapers of poly(3-hydroxybutyrate-*co*-3-hydroxyvalerate) (PHBV) containing oregano essential oil (OEO) and zinc oxide nanoparticles (ZnONPs).

Biopaper	L*	a*	b*	ΔE*	*T*	*O*
PHBV	89.82 ± 0.06 ^a^	0.87 ± 0.07 ^a^	−0.38 ± 0.02 ^a^	---	3.13 ± 0.02 ^a^	0.016 ± 0.06 ^a^
PHBV + 10 wt% OEO	85.35 ± 0.07 ^b^	1.13 ± 0.05 ^b^	6.67 ± 0.03 ^b^	8.36 ± 0.08 ^a^	3.55 ± 0.03 ^a^	0.019 ± 0.08 ^a^
PHBV + 3 wt% ZnONPs	88.11 ± 0.06 ^a^	0.56 ± 0.04 ^a^	−0.96 ± 0.05 ^c^	1.83 ± 0.05 ^b^	9.60 ± 0.06 ^b^	0.052 ± 0.05 ^b^
PHBV +2.5 wt% OEO +2.25 wt% ZnONPs	86.80 ± 0.03 ^c^	−0.92 ± 0.03 ^c^	4.32 ± 0.02 ^d^	5.87 ± 0.03 ^c^	8.08 ± 0.04 ^c^	0.041 ± 0.05 ^c^
PHBV + 5 wt% OEO + 1.5 wt% ZnONPs	84.10 ± 0.07 ^d^	−0.65 ± 0.06 ^d^	3.14 ± 0.09 ^e^	6.88 ± 0.09 ^d^	7.17 ± 0.05 ^d^	0.037 ± 0.05 ^c^
PHBV + 7.5 wt% OEO + 0.75 wt% ZnONPs	84.96 ± 0.06 ^bd^	−0.55 ± 0.08 ^e^	5.35 ± 0.06 ^f^	7.64 ± 0.08 ^e^	5.03 ± 0.06 ^e^	0.027 ± 0.05 ^d^

L*: Luminosity (+*L* luminous, −*L* dark), a*: red/green coordinates (+*a* red, −*a* green), b*: yellow/blue coordinates (+*b* yellow, −*b* blue), ΔE*: color difference, *T*: transparency, and *O*: Opacity. ^a–f^ Different letters in the same column indicate a significant difference (*p* < 0.05).

**Table 3:** Thermal properties of the zinc oxide nanoparticles (ZnONPs), oregano essential oil (OEO), and electrospun biopapers of poly(3-hydroxybutyrate-*co*-3-hydroxyvalerate) (PHBV) containing OEO and ZnONPs in terms of temperature at 5% mass loss (*T_5%_*), degradation temperature (*T_deg_*), mass loss at *T_deg_*, and residual mass at 700 °C.

Sample	*T_5%_* (°C)	*T_deg_* (°C)	Mass Loss (%)	Residual Mass (%)
OEO	101.5 ± 2.3	178.4 ± 0.89	74.16 ± 0.6	0.14 ± 0.5
ZnONPs	---	---	0.030 ± 0.5	99.97 ± 1.6
PHBV	251.5 ± 1.6	279.0 ± 1.1	55.23 ± 0.8	2.10 ± 0.7
PHBV + 10 wt% OEO	197.5 ± 2.7	283.6 ± 0.9	69.58 ± 1.0	2.16 ± 1.3
PHBV + 3 wt% ZnONPs	261.7 ± 2.3	275.6 ± 0.8	32.91 ± 1.4	4.35 ± 1.4
PHBV + 2.5 wt% OEO + 2.25 wt% ZnONPs	227.8 ± 2.1	261.4 ± 1.1	33.46 ± 0.7	3.77 ± 1.0
PHBV + 5 wt% OEO + 1.5 wt% ZnONPs	211.8 ± 1.3	263.3 ± 0.9	36.36 ± 1.9	3.44 ± 1.7
PHBV + 7.5 wt% OEO + 0.75 wt% ZnONPs	205.7 ± 1.9	268.9 ± 1.7	38.82 ± 1.5	3.15 ± 0.8

**Table 4:** Mechanical properties of the electrospun biopapers of poly(3-hydroxybutyrate-*co*-3-hydroxyvalerate) (PHBV) containing oregano essential oil (OEO) and zinc oxide nanoparticles (ZnONPs) in terms of tensile modulus (E), tensile strength at yield $\left(\sigma_{y}\right)$, elongation at break $\left(\varepsilon_{b}\right)$, and toughness.

Biopaper	*E* (MPa)	$\sigma_{y}$ (MPa)	$\varepsilon_{b}$ (%)	Toughness (mJ/mm^3^)
PHBV	1125 ± 441 ^a^	12.6 ± 2.7 ^a^	1.71 ± 0.35 ^a^	0.124 ± 0.005 ^a^
PHBV + 10 wt% OEO	814 ± 82 ^b^	18.5 ± 0.5 ^b^	4.53 ± 0.41 ^b^	0.560 ± 0.076 ^b^
PHBV + 3 wt% ZnONPs	1286 ± 142^c^	17.1 ± 0.9^c^	1.26 ± 0.23 ^c^	0.244 ± 0.048 ^c^
PHBV + 2.5 wt% OEO + 2.25 wt% ZnONPs	855 ± 118 ^d^	14.5 ± 2.4 ^d^	3.28 ± 0.32 ^d^	0.303 ± 0.061 ^d^
PHBV + 5 wt% OEO + 1.5 wt% ZnONPs	801 ± 93 ^b^	14.8 ± 4.5 ^d^	4.35 ± 1.28 ^b^	0.446 ± 0.273 ^e^
PHBV + 7.5 wt% OEO + 0.75 wt% ZnONPs	778 ± 102 ^e^	14.1 ± 3.8 ^d^	5.01 ± 1.34 ^e^	0.485 ± 0.249 ^e^

^a–e^ Different letters in the same column indicate a significant difference (*p* < 0.05).
